# Insufficient immune protection in preterm infants due to delayed or incomplete hexavalent vaccination

**DOI:** 10.3389/fimmu.2025.1626057

**Published:** 2025-10-21

**Authors:** Elisabeth Kaiser, Regine Weber, Michelle Bous, Ingmar Fortmann, Marie-Theres Dammann, Janina Marißen, Dorothee Viemann, Christoph Derouet, Steve Hein, Nasenien Nourkami-Tutdibi, Erol Tutdibi, Mara Wuhrmann, Muriel Charlotte Hans, Christian Gille, Stephan Gehring, Philipp Henneke, Christoph Härtel, Sybelle Goedicke-Fritz, Michael Zemlin

**Affiliations:** ^1^ Department of General Pediatrics and Neonatology, Saarland University, Homburg, Germany; ^2^ Department of Pediatrics, University Hospital of Lübeck, Lübeck, Germany; ^3^ Department of Pediatrics, University Hospital Würzburg, Würzburg, Germany; ^4^ Department of Neonatology, University of Heidelberg, Heidelberg, Germany; ^5^ Department of Pediatrics, University Medical Center Mainz, Mainz, Germany; ^6^ Department of Pediatrics, University of Freiburg, Freiburg, Germany

**Keywords:** preterm neonate, probiotics, microbiota, vaccination, randomized placebo-controlled clinical trial, antibody response, tetanus, diphtheria

## Abstract

**Introduction:**

For preterm infants, a 3 + 1 schedule is recommended for hexavalent vaccinations during the first year. The aim of this study was to analyze completion and timeliness of vaccinations in preterm infants of 28 + 0 – 32 + 6 weeks of gestation as part of the PRIMAL study (PRiming of IMmunity At the beginning of Life) and the antibody responses to vaccination antigens.

**Methods:**

Plasma antibody-concentrations against poliomyelitis, Haemophilus influenzae type b (Hib), diphtheria and tetanus were determined using ELISA and evaluated with respect to their protectiveness.

**Results:**

Among 82 patients that were recruited, paired plasma samples on admission and at the one year follow up visit were available in 41 infants. In 17 infants, plasma samples were also collected at two months, prior to the first vaccination. Transplacental antibody transfer yielded protective antibody concentrations against the vaccine antigens in 66% (Hib) to 93% (tetanus) of the infants on admission and in 24% (Polio) to 50% (diphtheria) at 2 months. At the one-year follow-up, all infants who received their vaccinations on time had complete immune protection. However, after one year, hexavalent vaccination was incomplete in 30 of 41 infants (73%). Among incompletely vaccinated infants, the proportion lacking protective antibody concentrations ranged from 12% for diphtheria to 27% for polio.

**Conclusions:**

Due to insufficient adherence to vaccination recommendations, 42% of highly vulnerable preterm neonates were insufficiently protected against one or more vaccine-preventable diseases after one year. Efforts should be increased to improve adherence to the recommended 3 + 1 vaccination schedule in preterm infants.

## 1 Introduction

Vaccination programs against vaccine-preventable diseases have significantly reduced child mortality and morbidity, making vaccines one of humanity’s greatest medical achievements (1). Multiple cohort studies have revealed that vaccinations are frequently delayed in preterm infants (2–7). Disturbingly, infants who were born very prematurely and likely to experience complicated clinical courses including prolonged mechanical ventilation were most likely to receive delayed vaccinations or none at all (4). In high-income countries, the main reason for non-adherence to recommended vaccination schedules is a lack of knowledge about the safety and efficiency of vaccinations on the part of healthcare professionals and parents (4, 8). Uncertainties regarding vaccination strategies appear to be particularly pronounced during the care for high-risk infants, such as very low birthweight infants (VLBW) and/or preterm neonates with bronchopulmonary dysplasia (4). However, it has been demonstrated that hexavalent and pentavalent vaccines (targeting diphtheria, tetanus, pertussis, poliomyelitis, Hib, and ± hepatitis B virus) are sufficiently immunogenic and have a favorable safety profile in preterm infants when administered in a 3 + 1 scheme (9, 10). For hexavalent and pneumococcus vaccinations, the German Standing Committee on Vaccination recommends a 2 + 1 scheme (2, 4 and 11 months) for term born and a 3 + 1 scheme (2, 3, 4 and 11 months) for preterm infants (11).

This study was conducted as part of the PRIMAL clinical trial and aimed to record the timeliness and completeness of the STIKO-recommended hexavalent vaccination of preterm infants of 28 + 0 – 32 + 6 weeks of gestation. Levels of plasma antibodies against poliomyelitis, Hib, diphtheria and tetanus were determined at the beginning of the second year of life and evaluated with respect to their protectiveness.

## 2 Materials and methods

### 2.1 Study design

This trial was part of PRIMAL (PRiming IMmunity At the beginning of Life), a double-blinded, randomized clinical control multi-center study addressing the safety and efficiency of a probiotic mix (Bifidobacterium (B.) animalis subsp. lactis =BB-12, B. longum subsp. infantis, Lactobacillus acidophilus = La-5; 5x109 CFUs/strain) in prevention of gut dysbiosis in VPN of 28 + 0 – 32 + 6 weeks of gestation (12, 13). The study medication was administered orally within 48 hours after birth and continued once daily for 28 days (**Supplementary Figure 1**). Venous blood was collected in EDTA tubes during routine venipunctures after study inclusion on day 1-3 (= admission), at 2 months (before the first vaccination) and at the one year follow-up (12 +/- 2 months). Blood samples were diluted 1:1 in phosphate-buffered saline, layered onto lymphocyte separation medium 1.077 (FicoLite-H, Linaris Biological Products, Dossenheim, Germany) and centrifuged at 300 × g for 15 minutes to separate plasma from mononuclear blood cells. Plasma was cryopreserved at -80 °C until processing.

### 2.2 ELISA

Enzyme-linked Immunosorbent Assays (ELISA) of IBL International GmbH, Hamburg, Germany were used to quantify plasma immunoglobulin G (IgG) levels against poliomyelitis virus (polio, ref. no. RE56921), polyribosylribitolphosphate of Haemophilus influenza type b (Hib, ref. no. RE56351), diphtheria toxin (DT, ref. no. 30113622) and tetanus toxoid (TT, ref. no. 30114074). All assays were performed according to the manufacturer’s instructions. In brief, plasma aliquots were thawed and prediluted prior to dispensing in triplicate onto the antigen-coated microtiter plates. Blanks, standards and controls were applied in parallel for measurements. HRP-detection antibody conjugates and TMB substrates indicated the presence of specific IgG. Absorbance was measured at 450/620 nm using microplate reader Infinite M Plex^®^ (Tecan Austria GmbH; Grödig, Austria). Standard curve was calculated as four-parameter logistic curve (GraphPad Prism version 9 for Windows, GraphPad Software, Boston, Massachusetts, USA). IgG plasma levels were compared with the threshold values specified by the ELISA manufacturer. Thresholds for protective (long-term) immunity: Polio IgG: ≥12 U/mL, Hib: ≥1 µg/mL, DT: ≥0.1 IU/mL, TT: ≥0.51 IU/mL. Thresholds for no protection: Polio IgG: <8 U/mL, Hib: <0.15 µg/mL, DT: <0.01 IU/mL, TT: <0.1 IU/mL.

### 2.3 Statistics

Statistical analysis was performed using SPSS (Chicago, USA) and graphs were generated using GraphPad Prism version 10 for Windows (GraphPad Software, Boston, Massachusetts, USA). Descriptive statistics show median and IQR unless stated otherwise. Mann-Whitney test was used to assess differences in quantitative data among independent groups. Wilcoxon matched-pairs signed-rank test was used to compare paired samples at admission, 2 months and one year. Fisher´s exact test was used to compare nominal variables. P-values were calculated using two-tailed tests, and the confidence level for statistical significance was set at 95%.

### 2.4 Ethics

The study was conducted in accordance with the Declaration of Helsinki and has been listed in the German Clinical Trials Register (DRKS-ID: DRKS00013197). The ethics committees (Ethikkommission bei der Ärztekammer des Saarlandes: 275_17; Ethik-Kommission der Universität zu Lübeck: 17_130) have approved the project. Parents of the participants gave written informed consent before being included in the study.

## 3 Results

### 3.1 Study population

**Figure 1:**
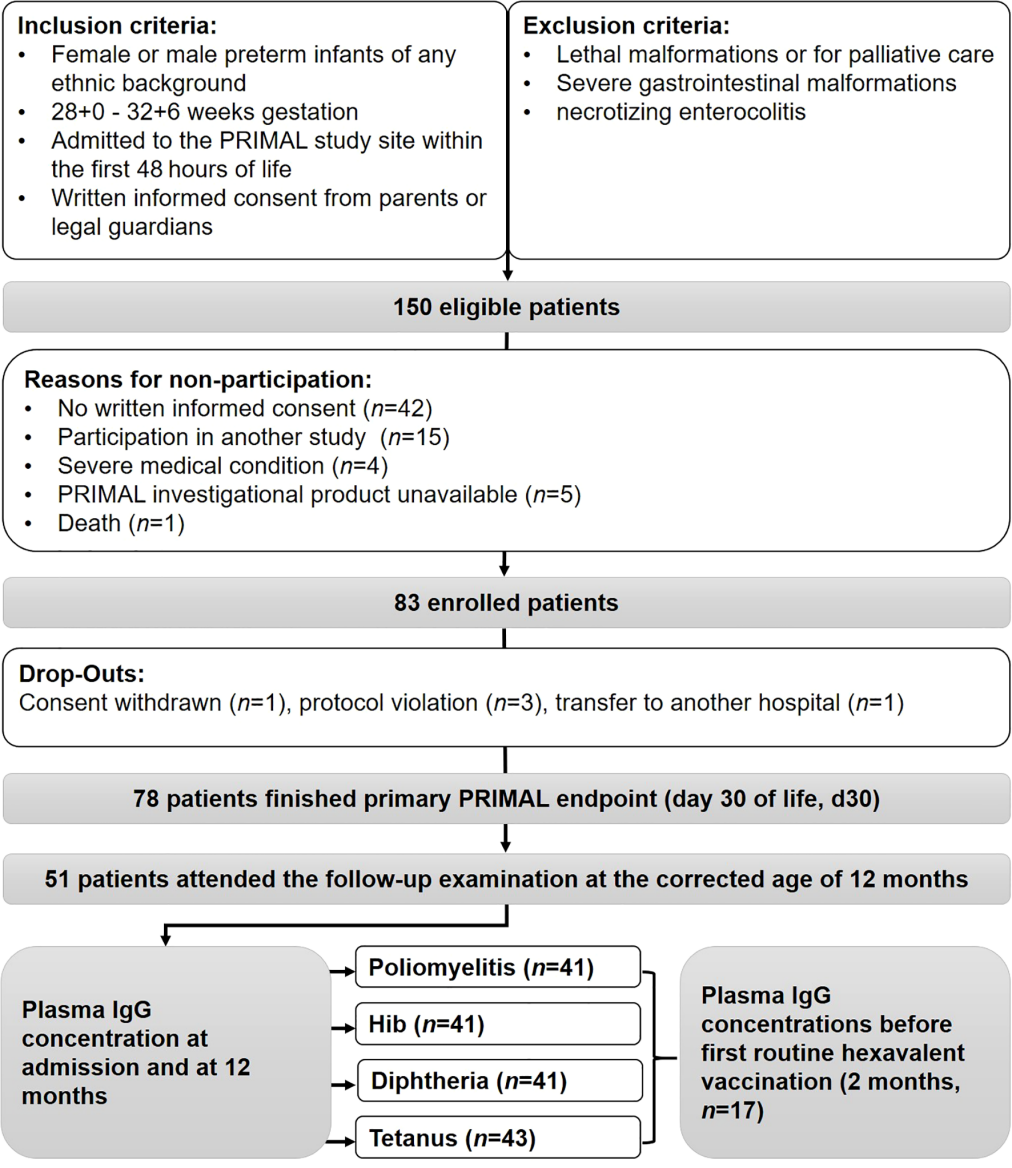
Trial recruitment and follow-up of study participants. IgG determination was available on admission and 12-months Plasma in 43 (tetanus) and 41 (poliomyelitis, Hib, diphtheria) participants, respectively.

A total of 150 infants born at the study site met the inclusion criteria and were screened (**Figure 1**). 83 children were included in the PRIMAL study, with one consent being withdrawn and four infants being excluded during the course of the study due to protocol violations or transfer to another hospital. Of the 78 remaining participants, 27 did not participate at the one year follow-up examination due to unavailability, unknown change of addresses, or restrictions associated with the Covid-19 pandemic. 51 attended the follow-up examination at the corrected age of one year. Plasma volumes sufficient for antibody measurement were sampled on admission and at one year from 41 participants (poliomyelitis, Hib, diphtheria) and 43 infants (tetanus toxoid), respectively. In 17 of these patients, plasma samples were also collected at the age of two months, prior to the first routine vaccination. In the remaining patients, blood samples could not be obtained at the age of two months due to discharge before first vaccination, insufficient blood volume or lack of clinical reason for venipuncture. The ethics approval restricted the study at 2 months of age to those infants that needed venipunctures for clinical reasons. Additional venipunctures exclusively for obtaining study samples were not allowed.

Detailed characteristics of the patients are given in **Supplementary Table 1a**. To identify potential bias due the availability of blood samples, we compared the cohorts of infants that were included in the study (83 minus one withdrawal of consent) with those infants of whom blood samples were available for analysis of all four vaccine specific IgG concentrations at 2 months (n=17) and at 12 months (n=41), respectively (**Supplementary Table 1b**). As expected, the group of infants that were still hospitalized during the first vaccination at 2 months had a lower birth weight (median 1.27 kg, Q1-Q3 1.24 kg-1.26 kg) compared to the infants that were included in the study (1.45 kg, Q1-Q3 1.25 kg-1.64 kg) and to the infants analysed at 12 months (1.46 kg, Q1-Q3 1.27 kg-1.62 kg) (p<0.05). The group that was studied at 12 months did not differ from the originally included patients regarding gestational age, sex distribution, and birth weight.

To compare the effectiveness of three versus four hexavalent vaccinations with regard to quantitative IgG levels against poliomyelitis, Hib, diphtheria and tetanus after one year, we grouped the participants according to the number of vaccinations received until the 12-month routine follow-up. These groups did not differ regarding gestational age, sex or postnatal age. Infants were vaccinated with either Infanrix hexa^®^ (GlaxoSmithKline, n=13) or Hexyon (Sanofi Pasteur^®^, n=12) or a combination of both (n=14). Regarding the vaccine used, no significant differences were found between the groups with three versus four vaccinations at the one-year follow-up. Being vaccinated only three times by the 12-month follow-up was associated with significantly later administration of the second, third and fourth hexavalent vaccinations in comparison to those infants who had received four vaccinations by the one-year follow up (**Table 1**).

**Table 1:** Characteristics of the study population by vaccination status at one year.

Variable	Unvaccinated at one year	3 vacc. at one year	4 vacc. at one year	*P* value (3 vs. 4 vacc. at one year)
*n*	3	28	11^b^	
Gestational Age in weeks, median (IQR)	32 3/7 (2 2/7)	30 5/7 (2 4/7)	29 5/7 (2 6/7)	0.99^d^
female, n (%)	2 (67)	16 (57)	4 (36)	0.301^e^
male, n (%)	1 (33)	12 (43)	7 (64)	
Birth weight, kg (IQR)	1.67 (0.42)	1.36 (0.24)	1.40 (0.68)	0.506^d^
Age at 1^st^ vaccination in days, median (IQR)	n.a.	87 (53)	65 (11)	0.076^d^
Age at 2^nd^ vaccination in days, median (IQR)	n.a.	146 (68)	105 (21)	**0.049** ^d^
Age at 3^rd^ vaccination in days, median (IQR)	n.a.	201 (87)	133 (30)	**0.003** ^d^
Age at 4^th^ vaccination in days, median (IQR)	n.a.	488 (98)	406 (61)^c^	**<0.0001** ^d^
Age at one year follow-up blood sampling in days: median (IQR)	437 (9)	438 (15)	444 (32)	0.206
Weight at one year follow-up, kg (IQR)	8.4 (2.0)	8.7 (2.3)	9.8 (1.4)	0.142^d^
Proportion *Hex* vaccinated (homologous), n (%)	n.a.	6 (21)	6 (55)	0.075^e^
Proportion *Ifx h* vaccinated (homologous), n (%)	n.a.	12 (43)	1 (9)	
Proportion *Hex* × *Ifx h* vaccinated (heterologous^a^), n (%)	n.a.	10 (36)	4 (36)	

vacc., vaccinations; Hex, Hexyon™ (Sanofi Pasteur); Ifx h, Infanrix hexa™ (GlaxoSmithKline); ^a^Since the two vaccines showed equal immunogenicity in our patients, they were not separated for analyses; ^b^Includes two individuals with tetanus IgG determination only; ^c^Two out of eleven individuals did not receive a fourth vaccination within the observation period of the study; ^d^Mann-Whitney test; ^e^Fisher´s exact test. Bold text: p< 0.05.

### 3.2 Timeliness and completeness of hexavalent vaccination

**Figure 2:**
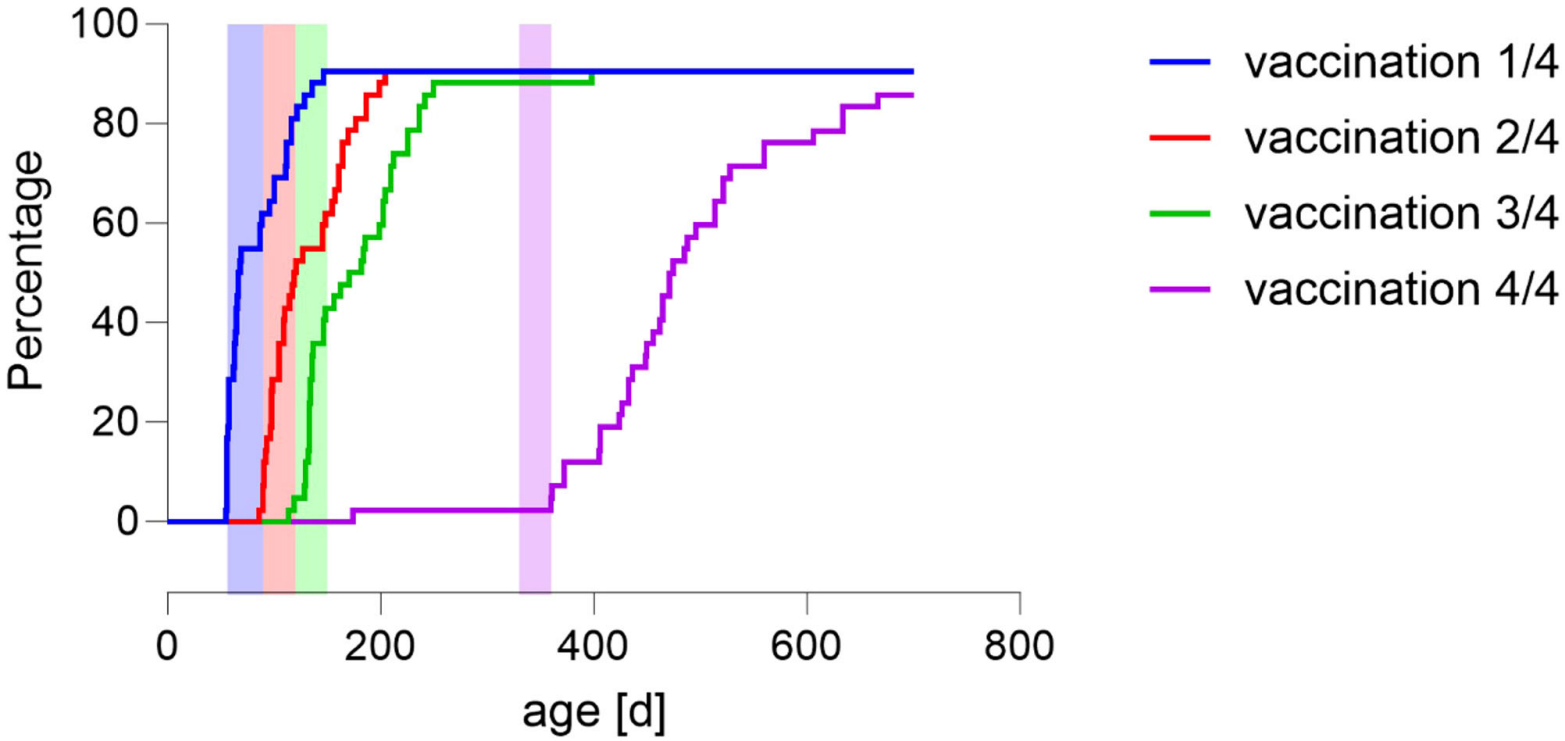
Time of vaccinations among the study population (n=42*) since one of the 43 participants was excluded due to missing information on vaccination status. The proportion of infants with delayed hexavalent vaccination was substantial. Kaplan-Meier curve demonstrates cumulative proportion of preterm infants receiving first (blue), second (red), third (green) and fourth (purple) hexavalent vaccinations to complete basic immunization as recommended by the German Standing Vaccination Committee for preterm infants. X-axis: preterm infants’ chronological age [d]. Shadowed vertical areas represent recommended vaccination age for 3 + 1 scheme (2, 3, 4, 11 months). Vertical dashed line shows median age at 12-months follow-up examination.

Vaccination records were examined for timeliness and completeness of hexavalent vaccination as recommended by the German Standing Vaccination Committee (**Figure 2**; **Supplementary Table 2**). In one infant the vaccination record was unavailable. Three participants remained unvaccinated until day 700. Of the remaining 39 participants, 25 infants received a timely first vaccination (2 months, cut-off: 90 days). Seventeen participants received the second vaccination (3 months, cut-off: 120 days), and 14 received the third vaccination (4 months, cut-off: 150 days) within the recommended timeframe. As a result, 59%, 40% and 33% of the study cohort received their first, second and third vaccinations on time, respectively. At day 700, two participants had received only three hexavalent vaccinations. All 37 remaining participants received the fourth vaccination six months or more after the third vaccination. Only two patients had completed the recommended 3 + 1 vaccination scheme at 11 months (360 and 361 days). On average the vaccination was completed at 485 days (16 months; n=37). The median time between the first and second vaccination was 42 days (13–99 days), 39 days between the second and the third vaccination (27–147 days) and 300 days between the third and fourth vaccination (190–474 days).

### 3.3 Course of IgG levels before and after hexavalent vaccination

**Figure 3:**
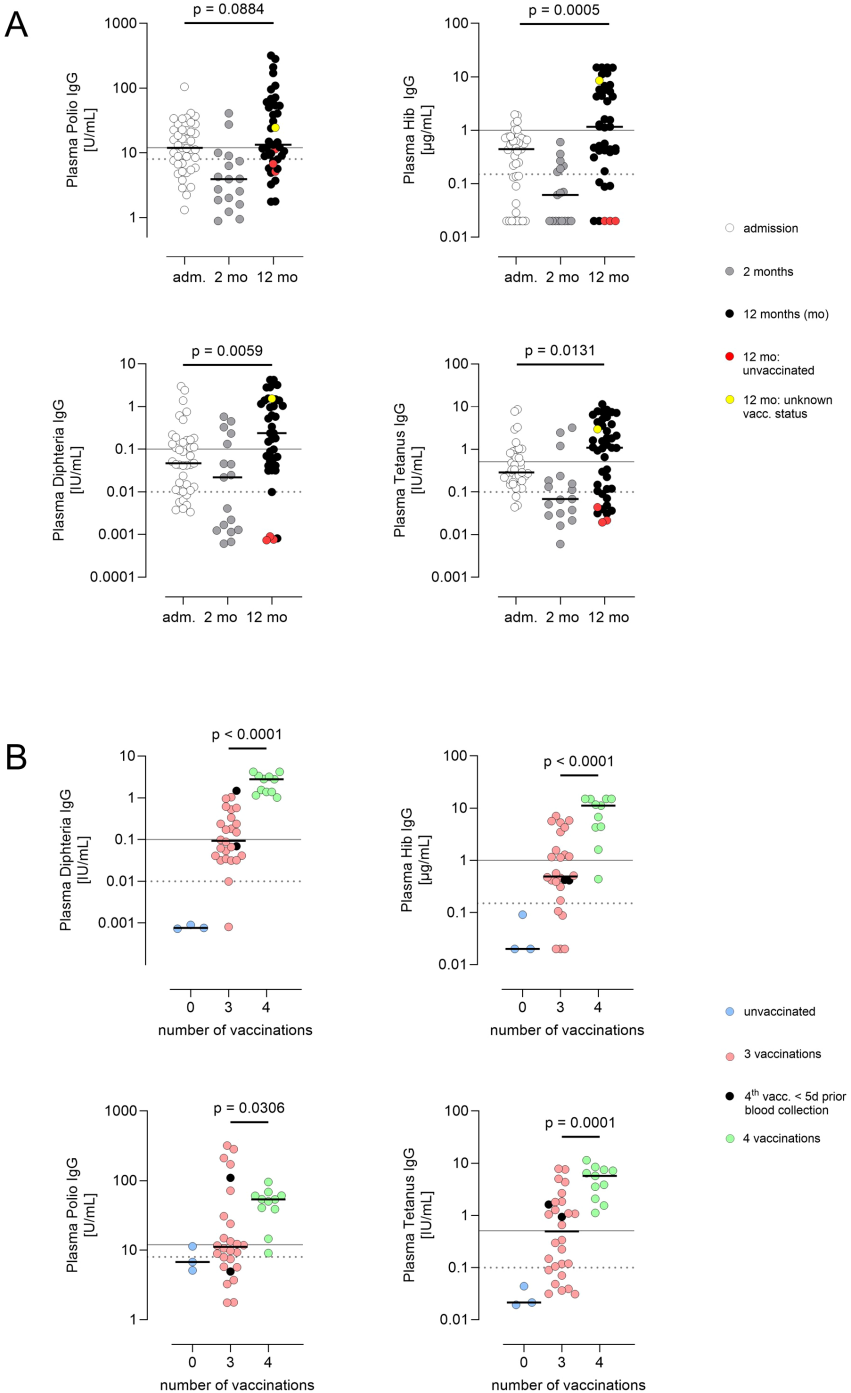
**(A)** Course of plasma IgG concentrations against polio, Hib, diphtheria and tetanus. All patients were tested after birth and at the corrected age of one year (n = 41 (polio, Hib, diphtheria) and 43 (tetanus)), and a subgroup of the cohort was also tested at 2 months before first hexavalent vaccination (n = 17). Adm.= admission, day 1 – 7; 2 mo = 2 months, prior to first routine hexavalent vaccination (day 52–96 of life); 12 mo = corrected age 12 +/- 2 months (day 357–505 of life). Red/yellow dots at one year: unvaccinated individuals/unknown vaccination status. Lines in scatter plots show median. P values were calculated using Wilcoxon matched-pairs signed rank test after exclusion of individuals with no vaccination or unknown vaccination status (n = 37 – 39). **(B)** Plasma IgG concentrations against polio, tetanus, Hib and diphtheria at one year depending on the number of vaccinations (12 +/- 2 months). Black dots: 4th vaccination administered, but <5 days prior to blood collection. Lines in scatter plots show medians. Solid line: threshold long-term protective immunity, dashed line: threshold no protection according to assay manufacturer’s specifications. Differences between children who received three and those who received four vaccine doses were calculated using the Mann–Whitney test. (n = 11 – 28).

As expected, maternally transferred antibody levels against tetanus toxoid, diphtheria toxoid, Haemophilus influenza type b (Hib) and poliomyelitis virus (polio) initially declined from birth until shortly before the first vaccination (tetanus at admission: 0.29 (0.56) IU/mL, 2 mo: 0.07 (0.18) IU/mL; diphtheria at admission: 0.05 (0.15) IU/mL, 2 mo: 0.02 (0.18) IU/mL; Hib at admission: 0.44 (0.65) mg/mL, 2 mo: 0.06 (0.18) mg/mL; polio at admission: 11.95 (16.4) U/mL, 2 mo: 3.93 (6.50) U/mL) (**Figure 3A**; **Supplementary Table 3**). Active immunization increased vaccination-specific antibody levels against tetanus, diphtheria, Hib and polio, whereby the increase between admission and 12 mo in the latter was not significant (p=0.088). Median (IQR) IgG levels at 12 mo of confirmed vaccinated individuals were determined as follows: tetanus 1.12 (4.21) IU/mL, diphtheria 0.24 (1.33) IU/mL, Hib 1.18 (5.31) µg/mL, polio 14.58 (52.18) U/mL. However, in a considerable proportion of vaccinated children, the immune response measured at the beginning of the second year of life was insufficient. Plasma IgG levels at 12 months are shown in **Figure 3B**, with results stratified by whether the 3 + 1 basic immunization scheme had been completed at the time of the follow-up analysis.

Two different licensed hexavalent vaccines were used (Hexyon™ and Infanrix hexa™). Both vaccines are approved by the German Standing Committee on Vaccination, contain the same active substances qualitatively (with one additional Bordetella antigen in Infanrix hexa™) and are equivalent in terms of immunogenicity (14). The analyses were made irrespective of the vaccine used, since no significant differences were found between the two vaccines.

### 3.4 Differences in IgG levels by number of vaccinations

Plasma IgG levels were found to be protective in the vast majority of those children already vaccinated four times at one year. Only one child had Hib IgG levels below the long-term protective concentration of 1 mg/µL, and one other child had marginally protective polio IgG levels. By contrast, in the triple-vaccinated group, 50% or more of the children did not reach long-term (tetanus, diphtheria, Hib) or robust (polio) protective levels in any of the four IgG titers estimated (IgG levels triple vaccinated at 12 mo: tetanus 0.50 (1.66) IU/mL, diphtheria 0.09 (0.34) IU/mL, Hib 0.49 (1.76) µg/mL, polio 11.13 (35.23 U/mL)). In some cases, the lower threshold for any protection (short-term immunity or non-solid immunity) was not reached (tetanus 7/28, diphtheria 2/26, Hib 5/26, polio 9/26). Quantitative differences between triple- and quadruple-vaccinated children were statistically significant in all cases.

### 3.5 Consideration of probiotic uptake

**Figure 4:**
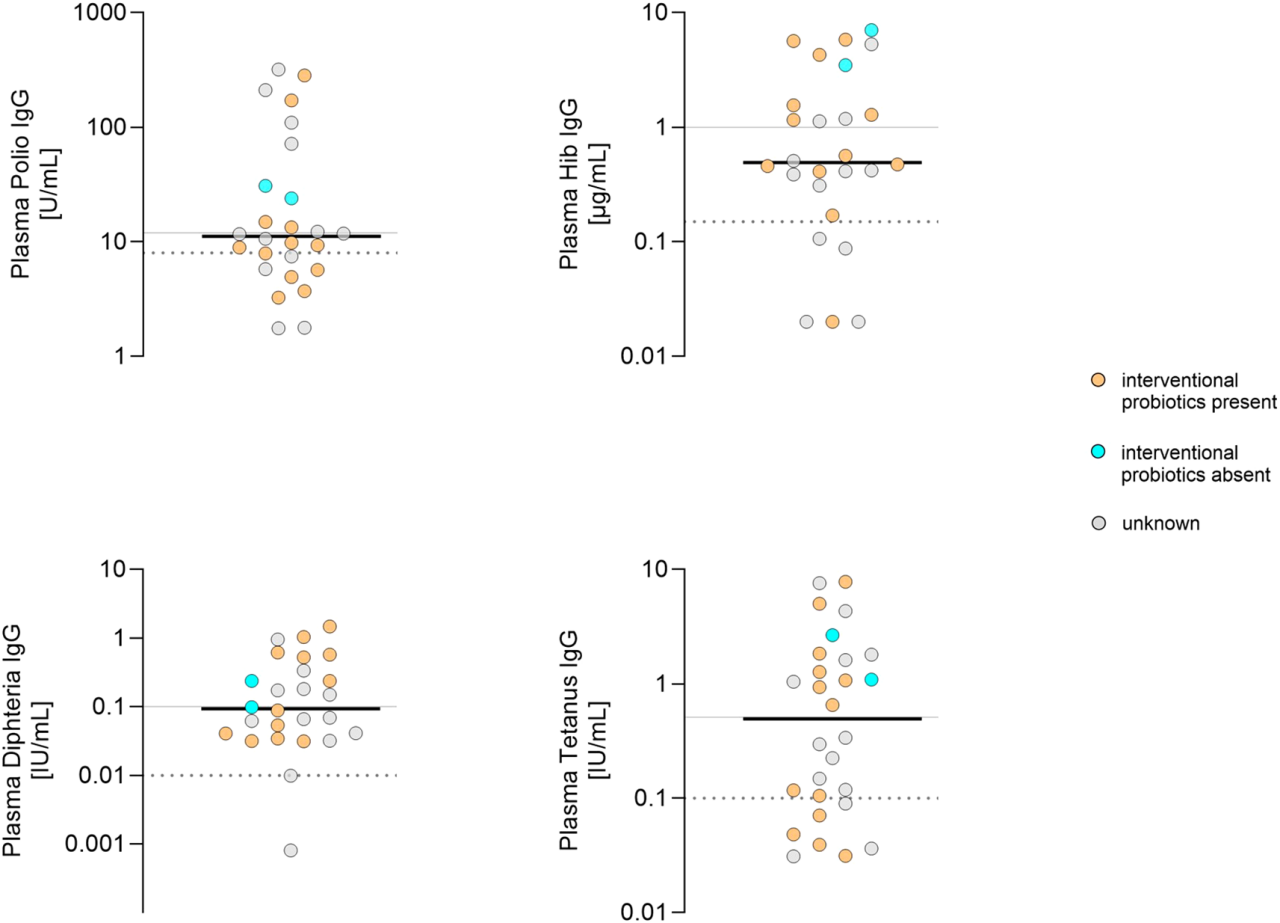
Plasma IgG concentrations at 12 months in infants that had obtained three vaccinations in relation to the presence of Bifidobacterium animalis subsp. lactis, B. infantis and/or Lactobacillus acidophilus in gut microbiome. Y- axis: plasma IgG levels of polio, Hib, diphtheria and tetanus. Cyan dots: No detection of Bifidobacterium animalis subsp. lactis, B. infantis and/or Lactobacillus acidophilus day 28 - 31. Orange dots: Bifidobacterium animalis subsp. lactis, B. infantis and/or Lactobacillus acidophilus present in gut microbiome on day 28 - 31. Grey dots: unknown gut microbiome status. Lines in scatter plots show median. Solid line: threshold long-term (tetanus, diphtheria, Hib) or robust (polio) protective immunity; dashed line: threshold no protection according to assay manufacturer’s specifications.

This study was conducted as a secondary analysis within the framework of the PRIMAL trial. In the triple-vaccinated group, analysis of 1 mo stool samples for PRIMAL-specific probiotic strains was possible in 53% of cases (**Figure 4**; **Supplementary Table 3**). In the majority, interventional probiotic strains Bifidobacterium animalis subsp. lactis, B. infantis and/or Lactobacillus acidophilus were detectable. Antibody titers did not differ between neonates with probiotic treatment or placebo. Thus, we found no significant probiotic effect on the immune response to vaccination.

## 4 Discussion

In this study, we found that the hexavalent vaccination (diphtheria, tetanus, pertussis, poliomyelitis, Hib, and hepatitis B) was delayed or incomplete at the 12-month follow-up visit in 73% of preterm infants with 28-32 + 6 weeks of gestation. At the 12-month follow-up all infants who were fully vaccinated according to the 3 + 1 schedule, as recommended by national guidelines, had protective antibody levels against diphtheria, tetanus, pertussis, poliomyelitis, and Hib. In contrast, only 45% of infants with incomplete vaccinations achieved protective antibody concentrations. The proportion of infants with long-term/robust protective immunity to all four vaccines was even lower (81% in fully vaccinated infants and 15% in infants with delayed vaccination). The proportion of infants experiencing a schedule delay increased with each of the four hexavalent primary immunizations. Thus, 42% of the highly vulnerable preterm neonates were unprotected against on or more vaccine-preventable disease after one year.

These findings underline that very preterm neonates require four vaccinations (3 + 1 vaccination scheme) during the first year of life to establish protective antibody titers against tetanus, diphtheria, Hib and poliomyelitis. By contrast, term neonates achieve protective antibody concentrations after three vaccinations (2 + 1 vaccination scheme (11, 15)). The majority of those preterm infants with initial basic immunization (three of four vaccinations) showed sufficient short-term immunity, which can be effectively boostered for long-term protection. Nevertheless, we observed that, depending on the antigen, 12% (diphtheria) to 27% (polio) of non-boostered infants showed no protective IgG levels at all.

For healthy term infants, the World Health Organization (WHO) recommends a 3 + 1 series of Diphtheria, Tetanus and Pertussis vaccines for all children beginning at 6 weeks (16). The WHO also recommends that national vaccination schedules should be adapted to the local epidemiology. Following a risk-benefit assessment, the German Standing Vaccination Committee adjusted its recommended hexavalent vaccination schedule for term born infants from a 3 + 1 (2, 3, 4, 11 months) schedule to a 2 + 1 (2, 4, 11 months) schedule (11, 17). However, the 3 + 1 schedule was retained for preterm infants since it was unknown whether a 2 + 1 regimen would yield sufficient protection in this cohort.

We found a high number of deviations from the recommended vaccination schedule in our study. Similar observations have been made in other countries, e.g. Israel (3) and Italy (18) and for other recommended vaccinations (19). In our study, 7% of the infants were not vaccinated at all due to a lack of parental consent. Even according to the 2 + 1 schedule recommended for term infants, one third of our preterm infants would still be affected by schedule delay. The most significant delay was observed for the final (fourth) booster vaccination, which, when administered at all, occurred between 190 and 474 days after the third dose.

Although the reasons for deviations from recommended vaccination schedules were not recorded, it is plausible that limited knowledge and vaccine hesitancy among both medical personnel and caregivers contributed to delays and incomplete vaccinations.

Public vaccination recommendations should clearly highlight exceptions to the general recommendations — particularly for high-risk patients such as preterm neonates. In the current guideline published by the German Standing Vaccination Committee, the 3 + 1 vaccination scheme for preterm neonates is included as one of fifteen footnotes to the recommendation for term infants, and these footnotes are printed in smaller font than the main document (20). We recommend that neonatologists should include the vaccination schedules for preterm neonates in discharge letters.

Approximately 2% of the German population have a negative attitude towards the routine vaccination of children, and 15% only partially agree with vaccination recommendations (21). Healthcare providers are the most trusted source of information on vaccinations and should therefore be enabled to provide patients with sufficient and up-to-date counseling (21, 22). Public information campaigns should be intensified to promote adherence to vaccination recommendations. The COVID-19 pandemic may have contributed to vaccination delays, as it coincided with the last months of the PRIMAL recruitment period and peaked with the public lockdown during the observation phase for vaccination schedule adherence. During this period, approximately 10% of the elective procedures, including vaccinations, were postponed (23–26). This is consistent with international observations (27–30).

Interestingly, a 2 + 1 vaccination schedule to Men B yielded protective antibody responses in preterm neonates, although antibody concentrations were lower compared to a 3 + 1 schedule (31). In contrast, we found that for hexavalent vaccines, a 2 + 1 vaccination schedule leaves many preterm neonates insufficiently protected. Thus, clinical trials are required to identify optimal vaccination schedules for each individual vaccine.

The lower immune response of preterm infants may originate from multiple factors. First, it is known that the primary (IgM) repertoire of preterm infants is characterized by a restricted use of VH, DH and JH gene segments and shorter immunoglobulin heavy chain third complementarity determining regions (CDR-H3) (32). Second, the secondary antibody repertoire (IgA and IgG) of preterm infants contains fewer somatic mutations (33, 34). Third, the antibody concentrations in response to vaccinations are lower in preterm neonates (35). These characteristics most likely reflect overlapping effects of the antigen-independent maturation of the fetal and neonatal immune system and the antigen-dependent stepwise establishment of secondary lymphatic structures and affinity maturation (33). In theory, the incomplete transfer of maternal vaccine-specific antibodies might be associated with a lower neutralization of vaccines in preterm neonates (36). However, this effect obviously does not compensate for the immaturity-associated quantitatively and qualitatively reduced response to vaccines in preterm neonates (35).

It is a limitation of the study that antibody concentrations only represent an estimate of immune protection, since antigen affinity is not assessed and may differ between individuals. Moreover, T-cell response significantly contributes to functional immune protection. In addition, it is difficult to predict whether the immune response of a given individual to a wildtype infection is associated with an asymptomatic carrier status or not. Various publications demonstrate a correlation between the antibody concentrations that convey short term protection (presumably achieved by a set of primary vaccinations) and long-term protection (presumably achieved by booster vaccination) (37, 38). The subgroup of infants that were available to antibody analysis at 2 months was less mature than the total study group, thus we cannot rule out a bias by greater immaturity in this group of infants. Furthermore, the study needs to be interpreted carefully, since the incompletely vaccinated patients were a heterogeneous group: Some of the incompletely vaccinated patients were vaccinated according to a 2 + 1 schedule, others according to a 3 + 0 schedule and a third group was vaccinated at irregular time points. Finally, the PRIMAL study revealed a frequent nosocomial transmission of probiotics and thus (12, 13), the study was most likely underpowered to assess the influence of probiotics on the vaccine-specific immune response (39).

To our knowledge, this is the first study to assess antibody concentrations for four components of the hexavalent vaccines in cord blood and peripheral blood, with a one-year follow-up in a cohort of preterm infants of 28–32 weeks. A key strength of our study is the measurement of the concentration of maternal antibodies (cord blood and prior to first immunization), allowing an estimate of the preterm neonate’s own antibody production (after first vaccination and at the one-year follow-up).

## 5 Conclusion

Vaccination delays or omissions affect the majority of preterm infants and result in insufficient protection against vaccine-preventable diseases in this highly susceptible population. Preterm neonates should receive hexavalent vaccinations according to the 3 + 1 schedule during the first year, since the 2 + 1 schedule recommended for healthy term infants leaves 55% of preterm infants insufficiently protected. This is particularly relevant, as our data indicate that the timely administration of the booster vaccination is crucial for ensuring children are protected when they enter early daycare. Thus, the immaturity of the adaptive immune response of preterm neonates has direct clinical consequences. Authorities should emphasize that vaccination recommendations for preterm neonates (and other high-risk groups) may differ from those for healthy infants. Information campaigns on vaccination recommendations addressing healthcare workers and parents should be intensified. Further studies should be undertaken to optimize vaccines, adjuvants and vaccination schedules in high-risk patients such as preterm neonates.

## Data Availability

The original contributions presented in the study are included in the article/**Supplementary Material**. Further inquiries can be directed to the corresponding author.

## Supplementary material

Supplementary Figure 1 Sampling schedule for the present trial as part of the PRIMAL study [Marißen et al., 2019, Van Rossum et al., 2024]. Blood samples were collected **(A)** after admission (median 3 days of life, IQR 1 day), **(C)** two months after birth prior to the first hexavalent vaccination (median 56 days of life, IQR 10 days) and **(D)** at one year follow up = corrected age of approximately 12 months (median 437 days of life, IQR 24 days). Stool samples for microbiome analysis were collected **(B)** at one month (median 30 days of life, IQR 2 days). The PRIMAL trial intervention (blue bar) started as soon as possible after randomization with daily enteral administration of the probiotic mix (Bifidobacterium longum, B. infantis and Lactobacillus acidophilus) or placebo over 28 days. • Marissen J, Haiss A, Meyer C, et al. Efficacy of Bifidobacterium longum, B. infantis and Lactobacillus acidophilus probiotics to prevent gut dysbiosis in preterm infants of 28 + 0-32 + 6 weeks of gestation: a randomised, placebo-controlled, double-blind, multicentre trial: the PRIMAL Clinical Study protocol. BMJ Open. 2019;9:e032617 doi: 10.1136/bmjopen-2019-032617. • Van Rossum T, Haiss A, Knoll RL, et al. Bifidobacterium and Lactobacillus Probiotics and Gut Dysbiosis in Preterm Infants: The PRIMAL Randomized Clinical Trial. JAMA Pediatr. 2024;178:985–95 doi: 10.1001/jamapediatrics.2024.2626.
