# Supplementary material for: Insufficient immune protection in preterm infants due to delayed or incomplete hexavalent vaccination
Source: Front Immunol. 2025 Oct 21;16:1626057. doi: 10.3389/fimmu.2025.1626057 (PMC12583041; doi:10.3389/fimmu.2025.1626057)
Supplement: Supplementary Figure 1 — Sampling schedule for the present trial as part of the PRIMAL study [Marißen et al., 2019, Van Rossum et al., 2024]. Blood samples were collected (A) after admission (median 3 days of life, IQR 1 day), (C) two months after birth prior to the first hexavalent vaccination (median 56 days of life, IQR 10 days) and (D) at one year follow up = corrected age of approximately 12 months (median 437 days of life, IQR 24 days). Stool samples for microbiome analysis were collected (B) at one month (median 30 days of life, IQR 2 days). The PRIMAL trial intervention (blue bar) started as soon as possible after randomization with daily enteral administration of the probiotic mix (Bifidobacterium longum, B. infantis and Lactobacillus acidophilus) or placebo over 28 days. • Marissen J, Haiss A, Meyer C, et al. Efficacy of Bifidobacterium longum, B. infantis and Lactobacillus acidophilus probiotics to prevent gut dysbiosis in preterm infants of 28 + 0-32 + 6 weeks of gestation: a randomised, placebo-controlled, double-blind, multicentre trial: the PRIMAL Clinical Study protocol. BMJ Open. 2019;9:e032617 doi: 10.1136/bmjopen-2019-032617. • Van Rossum T, Haiss A, Knoll RL, et al. Bifidobacterium and Lactobacillus Probiotics and Gut Dysbiosis in Preterm Infants: The PRIMAL Randomized Clinical Trial. JAMA Pediatr. 2024;178:985–95 doi: 10.1001/jamapediatrics.2024.2626. [file Table1.docx]

Supplementary Material

Insufficient immune protection in preterm infants due to delayed and incomplete hexavalent vaccination

Elisabeth Kaiser^1^, Regine Weber^1^, Michelle Bous^1^, Ingmar Fortmann^2^, Marie-Theres Dammann^2^, Janina Marißen^3^, Dorothee Viemann^3^, Christoph Derouet^1^, Steve Hein^1^, Nasenien Nourkami-Tutdibi^1^, Erol Tutdibi^1^, Mara Wuhrmann^1^, Muriel Charlotte Hans^1^, Christian Gille^4^, Dorothee Viemann^3^, Stephan Gehring^5^, Philipp Henneke^6^, Christoph Härtel^3^, Sybelle Goedicke-Fritz^1^, Michael Zemlin^1^* For the PRIMAL clinical study consortium

1 Department of General Pediatrics and Neonatology, Saarland University, 66123 Homburg, Germany

2 Department of Pediatrics, University Hospital of Lübeck, 23538 Lübeck, Germany

3 Department of Pediatrics, University Hospital Würzburg, Würzburg, Germany

4 Department of Neonatology, University of Heidelberg, Heidelberg, Germany

5 Department of Pediatrics, University Medical Center Mainz, Mainz, Germany

6 Department of Pediatrics, University of Freiburg, Freiburg, Germany

*** Correspondence:**corresponding author: michael.zemlin@uks.eu

# Supplementary Tables

**Supplementary Table 1a**. Raw data for Figure 1 and Table 1.

| **Patient ID** | **Sex** | **Gestational age (weeks + days)** | **Gestational age (d)** | **multiple birth** | **Age vacc. 1 (d)** | **Age vacc. 2 (d)** | **Age vacc. 3 (d)** | **Age vacc. 4 (d)** | **Vaccine used** | **Age at 12 mo follow-up (d)** | **Birth weight [kg]** | **12 mo follow-up weight [kg]** | **Titer determ.: adm. + 12 mo** | **Titer determ.: 2 mo** |
| --- | --- | --- | --- | --- | --- | --- | --- | --- | --- | --- | --- | --- | --- | --- |
| **12.1.8.001** | f | 31+3 | 220 | twins | 136 | 170 | 204 | 488 | Hex x Ifx h | 446 | 1.9 | 11.4 | yes | no |
| **12.2.7.001** | f | 29+0 | 203 | twins | 58 | 146 | 186 | 475 | Ifx h | 440 | 1.085 | 9.3 | yes | yes |
| **12.2.8.002** | f | 32+3 | 227 | no | 66 | 127 | 163 | 360 | Hex | 423 | 1.8 | 9.5 | yes | no |
| **12.1.7.001** | m | 30+1 | 211 | twins # | 116 | 161 | 226 | 471 | Hex | 424 | 1.485 | 9.0 | yes | no |
| **12.1.7.002** | m | 30+1 | 211 | twins # | 116 | 161 | 226 | 471 | Hex | 424 | 1.45 | 8.4 | yes | no |
| **12.2.7.003** | f | 30+5 | 215 | twins # | 56 | 90 | 130 | 560 | Ifx h | 458 | 1.25 | 11.5 | yes | yes |
| **12.2.7.004** | f | 30+5 | 215 | twins # | 56 | 90 | 130 | 560 | Ifx h | 459 | 1.48 | 10.4 | yes | yes |
| **12.2.8.003** | f | 31+6 | 223 | no | 64 | 117 | 148 | 456 | Hex | 417 | 1.475 | 8.2 | yes | no |
| **12.1.7.005** | m | 29+3 | 206 | no | 147 | 204 | 398 | n/a | Ifx h | 439 | 1.3 | 10.2 | yes | no |
| **12.2.8.005** | f | 31+6 | 223 | no | 62 | 97 | 132 | 606 | Ifx h | 431 | 1.65 | 8.2 | yes | no |
| **12.2.8.006** | f | 32+3 | 227 | quadruplets # | 87 | 199 | 250 | 634 | Hex x Ifx h | 432 | 1.36 | 7.7 | yes | no |
| **12.2.8.007** | f | 32+3 | 227 | quadruplets # | 87 | 187 | 236 | 634 | Hex x Ifx h | 432 | 1.605 | 8.1 | yes | no |
| **12.1.8.008** | m | 32+3 | 227 | quadruplets # | 89 | 187 | 236 | 667 | Hex x Ifx h | 432 | 1.57 | 7.9 | yes | no |
| **12.1.8.009** | m | 32+3 | 227 | quadruplets # | 89 | 184 | 243 | 667 | Hex x Ifx h | 432 | 1.405 | 8.3 | yes | no |
| **12.1.8.010** | m | 32+4 | 228 | no | 65 | 93 | 136 | 424 | Hex | 444 | 2.02 | 9.4 | yes | no |
| **12.1.7.012** | m | 29+1 | 204 | twins # | 69 | 109 | 210 | 465 | Ifx h | 446 | 1.24 | 10.4 | yes | yes |
| **12.2.7.009** | f | 29+1 | 204 | twins # | 96 | 109 | 210 | 465 | Ifx h | 446 | 1.155 | 7.6 | yes | yes |
| **12.2.7.011** | f | 28+2 | 198 | no | 56 | 119 | 171 | 427 | Hex x Ifx h | 443 | 1.27 | 9.0 | yes | yes |
| **12.1.8.011** | m | 32+3 | 227 | twins # | n/a | n/a | n/a | n/a | none | 437 | 2.055 | 9.8 | yes | no |
| **12.2.8.013** | f | 32+3 | 227 | twins # | n/a | n/a | n/a | n/a | none | 437 | 1.64 | 8.4 | yes | no |
| **12.2.8.014** | f | 30+5 | 215 | twins # | 112 | 164 | 203 | 514 | Ifx h | 478 | 1.21 | 10.9 | yes | no |
| **12.2.8.015** | f | 30+5 | 215 | twins # | 112 | 164 | 203 | 514 | Ifx h | 478 | 1.35 | 10.5 | yes | no |
| **12.1.7.015** | m | 29+5 | 208 | twins # | 67 | 105 | 133 | 433 | Hex x Ifx h | 456 | 1.245 | 10.0 | yes | yes |
| **12.1.7.016** | m | 29+5 | 208 | twins # | 67 | 105 | 133 | 433 | Hex x Ifx h | 456 | 1.24 | 9.2 | yes | yes |
| **12.2.8.016** | f | 32+4 | 228 | no | 122 | 148 | 182 | 372 | Hex | 435 | 2.07 | n/a | yes | no |
| **12.1.7.017** | m | 29+5 | 208 | twins # | 56 | 98 | 133 | 406 | Hex | 434 | 1.099 | 10.5 | yes | yes |
| **12.1.7.018** | m | 29+5 | 208 | twins # | 56 | 98 | 133 | 406 | Hex | 434 | 1.3 | 11.1 | yes | yes |
| **12.2.7.012** | f | 29+2 | 205 | twins # | 63 | 110 | 147 | 522 | Hex x Ifx h | 440 | 1.25 | 7.4 | yes | yes |
| **12.1.7.019** | m | 29+2 | 205 | twins # | 63 | 115 | 147 | 522 | Hex | 440 | 1.495 | 10.4 | yes | yes |
| **12.1.8.016** | m | 32+6 | 230 | no | 111 | 146 | 184 | 405 | Hex | 410 | 1.3 | 8.0 | yes | no |
| **12.1.8.018** | m | 30+1 | 211 | no | n/a | n/a | n/a | n/a | none | 428 | 1.665 | 7.8 | yes | no |
| **12.1.8.019** | m | 32+3 | 227 | no | 65 | 99 | 129 | 372 | Hex | 472 | 1.92 | 10.0 | yes | no |
| **12.2.7.013** | f | 29+2 | 205 | twins | 58 | 105 | 134 | 449 | Ifx h | 505 | 1.4 | 7.8 | yes | yes |
| **12.2.7.015** | f | 28+4 | 200 | no | 56 | 98 | 137 | 539 | Ifx h | 436 | 1.24 | 8.6 | yes | no |
| **12.2.7.016** | f | 30+4 | 214 | no | 100 | 155 | 242 | 528 | Hex x Ifx h | 484 | 1.485 | 9.2 | yes | no |
| **12.2.8.017** | f | 32+1 | 225 | no | 129 | 157 | 199 | n/a | Ifx h | 474 | 1.435 | 8.3 | yes | no |
| **12.2.7.017** | f | 30+1 | 211 | twins # | 58 | 91 | 136 | 462 | Hex x Ifx h | 437 | 1.27 | 9.8 | yes | yes |
| **12.1.7.024** | m | 30+1 | 211 | twins # | 58 | 91 | 134 | 450 | Hex x Ifx h | 437 | 1.34 | 8.7 | yes | yes |
| **12.1.7.025** | m | 29+4 | 207 | no | 57 | 92 | 119 | 361 | Hex x Ifx h | 465 | 1.485 | 10.5 | yes | yes |
| **12.1.7.026** | m | 29+4 | 207 | no | 68 | 121 | 156 | 436 | Ifx h | 439 | 1.36 | 8.7 | yes | yes |
| **12.2.8.020** | f | 31+3 | 220 | no | n/a | n/a | n/a | n/a | unknown | 433 | 1.765 | 9.4 | yes | no |
| **12.2.8.021** | f | 31+4 | 221 | no | 100 | 177 | 212 | 485 | Hex | 425 | 1.64 | 9.1 | yes* | no |
| **12.1.7.027** | m | 28+3 | 199 | no | 55 | 86 | 114 | 496 | Hex x Ifx h | 357 | 1.36 | 8.7 | yes* | no |
| 12.1.8.004 | m | 32+5 | 229 | twins | unknown | unknown | unknown | unknown | unknown | n/a | 1.64 | n/a | no | no |
| 12.2.8.004 | m | 32+5 | 229 | twins | unknown | unknown | unknown | unknown | unknown | n/a | 1.08 | n/a | no | no |
| 12.1.8.002 | m | 32+6 | 230 | no | unknown | unknown | unknown | unknown | unknown | 425 | 1.855 | 8.9 | no | no |
| 12.2.7.002 | f | 29+4 | 207 | no | unknown | unknown | unknown | unknown | unknown | n/a | 1.24 | n/a | no | no |
| 12.1.8.005 | m | 32+1 | 225 | no | unknown | unknown | unknown | unknown | unknown | n/a | 1.25 | n/a | no | no |
| 12.1.8.006 | m | 32+3 | 227 | no | unknown | unknown | unknown | unknown | unknown | n/a | 1.9 | n/a | no | no |
| 12.1.8.007 | m | 32+3 | 227 | no | unknown | unknown | unknown | unknown | unknown | 405 | 0.98 | 6.3 | no | no |
| 12.1.7.003 | m | 28+2 | 198 | no | unknown | unknown | unknown | unknown | unknown | n/a | 1.2 | n/a | no | no |
| 12.1.7.004 | m | 28+2 | 198 | no | unknown | unknown | unknown | unknown | unknown | n/a | 1.205 | n/a | no | no |
| 12.2.7.005 | f | 30+0 | 210 | no | unknown | unknown | unknown | unknown | unknown | n/a | 1.475 | n/a | no | no |
| 12.1.7.006 | m | 30+3 | 213 | no | unknown | unknown | unknown | unknown | unknown | n/a | 1.66 | n/a | no | no |
| 12.1.7.007 | f | 30+5 | 215 | no | unknown | unknown | unknown | unknown | unknown | n/a | 1.61 | n/a | no | no |
| 12.1.7.008 | m | 30+3 | 213 | no | unknown | unknown | unknown | unknown | unknown | n/a | 2.03 | n/a | no | no |
| 12.2.7.006 | f | 29+3 | 206 | no | unknown | unknown | unknown | unknown | unknown | n/a | 1.4 | n/a | no | no |
| 12.2.7.007 | f | 29+5 | 208 | twins | unknown | unknown | unknown | unknown | unknown | n/a | 1.32 | n/a | no | no |
| 12.2.7.008 | f | 29+5 | 208 | twins | unknown | unknown | unknown | unknown | unknown | n/a | 1.47 | n/a | no | no |
| 12.2.8.008 | f | 32+1 | 225 | no | unknown | unknown | unknown | unknown | unknown | n/a | 1.81 | n/a | no | no |
| 12.2.8.009 | f | 30+6 | 216 | no | unknown | unknown | unknown | unknown | unknown | n/a | 1.96 | n/a | no | no |
| 12.1.7.009 | m | 30+3 | 213 | no | unknown | unknown | unknown | unknown | unknown | n/a | 1.2 | n/a | no | no |
| 12.2.8.010 | f | 32+6 | 230 | triplets | unknown | unknown | unknown | unknown | unknown | n/a | 1.45 | n/a | no | no |
| 12.2.8.011 | f | 32+6 | 230 | triplets | unknown | unknown | unknown | unknown | unknown | n/a | 1.715 | n/a | no | no |
| 12.2.8.012 | f | 32+6 | 230 | triplets | unknown | unknown | unknown | unknown | unknown | n/a | 1.87 | n/a | no | no |
| 12.1.7.010 | m | 28+4 | 200 | twins | unknown | unknown | unknown | unknown | unknown | n/a | 1.175 | n/a | no | no |
| 12.1.7.011 | m | 28+4 | 200 | twins | unknown | unknown | unknown | unknown | unknown | n/a | 1.22 | n/a | no | no |
| 12.2.7.010 | f | 30+6 | 216 | no | unknown | unknown | unknown | unknown | unknown | 424 | 1.34 | 7.0 | no | no |
| 12.1.7.013 | m | 30+6 | 216 | no | unknown | unknown | unknown | unknown | unknown | n/a | 1.635 | n/a | no | no |
| 12.1.7.014 | m | 28+4 | 200 | no | unknown | unknown | unknown | unknown | unknown | 490 | 1.24 | 11.3 | no | no |
| 12.1.8.012 | m | 30+2 | 212 | no | unknown | unknown | unknown | unknown | unknown | 494 | 1.468 | 10.9 | no | no |
| 12.1.8.013 | m | 32+6 | 230 | no | unknown | unknown | unknown | unknown | unknown | n/a | 1.42 | n/a | no | no |
| 12.1.8.014 | m | 31+3 | 220 | no | unknown | unknown | unknown | unknown | unknown | n/a | 1.195 | n/a | no | no |
| 12.1.8.015 | m | 32+1 | 225 | no | unknown | unknown | unknown | unknown | unknown | n/a | 1.35 | n/a | no | no |
| 12.2.8.018 | f | 32+3 | 227 | no | unknown | unknown | unknown | unknown | unknown | n/a | 1.845 | n/a | no | no |
| 12.1.7.020 | m | 30+3 | 213 | no | unknown | unknown | unknown | unknown | unknown | n/a | 1.475 | n/a | no | no |
| 12.1.8.020 | m | 32+5 | 229 | no | unknown | unknown | unknown | unknown | unknown | 477 | 1.64 | 11.5 | no | no |
| 12.1.7.021 | m | 30+3 | 213 | no | unknown | unknown | unknown | unknown | unknown | 463 | 1.94 | 11.9 | no | no |
| 12.1.7.022 | m | 29+2 | 205 | twins | unknown | unknown | unknown | unknown | unknown | 505 | 1.44 | 10.2 | no | no |
| 12.2.7.014 | f | 29+5 | 208 | no | unknown | unknown | unknown | unknown | unknown | n/a | 1.155 | n/a | no | no |
| 12.2.8.019 | f | 31+0 | 217 | no | unknown | unknown | unknown | unknown | unknown | n/a | 1.495 | n/a | no | no |
| 12.1.8.025 | m | 31+5 | 222 | twins | unknown | unknown | unknown | unknown | unknown | n/a | 1.845 | n/a | no | no |

Bold written patient-IDs indicate individuals who were included in the titer determination.

#: titer determination in both twins/ all four quadruplets

*: Tetanus IgG determination only

**Supplementary Table 1b**. Characteristics of patient that completed the study until discharge, patients with complete analysis of vaccine-specific IgG before first vaccination and patients with complete analysis of vaccine-specific IgG at 12 months follow-up.

| **Characteristic** | **Patients that completed the study until discharge (n=82)** | **Patients with complete analysis of vaccine specific IgG before first vaccination (n=17)** | **Patients with complete analysis of vaccine-specific IgG at 12 months follow-up (n=41)** |
| --- | --- | --- | --- |
| Gestational age, weeks + days. Median (Q1-Q3) | 30 5/7  (29 5/7-32 3/7) | 29 4/7  (29 3/7-29 5/7) | 30 4/7  (29 4/7-32 3/7) |
| Birth weight, kg median (Q1-Q3) | 1.45  (1.25-1.64) | 1.27  (1.24-1.36) | 1.41  (1.27-1.62) |
| Female, n, (%) | 38 (46%) | 8 (47%) | 23 (56%) |

**Supplementary Table 2**. Raw data for Figure 2

| **Day of vaccination^1^** | **vaccination 1**  No of infants | **vaccination 2**  No of infants | **vaccination 3**  No of infants | **vaccination 4**  No of infants |
| --- | --- | --- | --- | --- |
| 0 | 42 | 42 | 42 | 42 |
| 55 | 42 |  |  |  |
| 56 | 41 |  |  |  |
| 57 | 35 |  |  |  |
| 58 | 34 |  |  |  |
| 62 | 30 |  |  |  |
| 63 | 29 |  |  |  |
| 64 | 27 |  |  |  |
| 65 | 26 |  |  |  |
| 66 | 24 |  |  |  |
| 67 | 23 |  |  |  |
| 68 | 21 |  |  |  |
| 69 | 20 |  |  |  |
| 86 |  | 42 |  |  |
| 87 | 19 |  |  |  |
| 89 | 17 |  |  |  |
| 90 |  | 41 |  |  |
| 91 |  | 39 |  |  |
| 92 |  | 37 |  |  |
| 93 |  | 36 |  |  |
| 96 | 15 |  |  |  |
| 97 |  | 35 |  |  |
| 98 |  | 34 |  |  |
| 99 |  | 31 |  |  |
| 100 | 14 |  |  |  |
| 105 |  | 30 |  |  |
| 109 |  | 27 |  |  |
| 110 |  | 25 |  |  |
| 111 | 12 |  |  |  |
| 112 | 11 |  |  |  |
| 114 |  |  | 42 |  |
| 115 |  | 24 |  |  |
| 116 | 9 |  |  |  |
| 117 |  | 23 |  |  |
| 119 |  | 22 | 41 |  |
| 121 |  | 21 |  |  |
| 122 | 7 |  |  |  |
| 127 |  | 20 |  |  |
| 129 | 6 |  | 40 |  |
| 130 |  |  | 39 |  |
| 132 |  |  | 37 |  |
| 133 |  |  | 36 |  |
| 134 |  |  | 32 |  |
| 136 | 5 |  | 30 |  |
| 137 |  |  | 28 |  |
| 146 |  | 19 |  |  |
| 147 |  |  | 27 |  |
| 148 |  | 17 | 25 |  |
| 155 |  | 16 |  |  |
| 156 |  |  | 24 |  |
| 157 |  | 15 |  |  |
| 161 |  | 14 |  |  |
| 163 |  |  | 23 |  |
| 164 |  | 12 |  |  |
| 170 |  | 10 |  |  |
| 171 |  |  | 22 |  |
| 177 |  | 9 |  |  |
| 182 |  |  | 21 |  |
| 184 |  | 8 | 20 |  |
| 186 |  |  | 19 |  |
| 187 |  | 7 |  |  |
| 199 |  | 5 | 18 |  |
| 203 |  |  | 17 |  |
| 204 |  | 4 | 15 |  |
| 210 |  |  | 14 |  |
| 212 | 4 |  | 12 |  |
| 226 |  |  | 11 |  |
| 236 |  |  | 9 |  |
| 242 |  |  | 7 |  |
| 243 |  |  | 6 |  |
| 250 |  |  | 5 |  |
| 360 |  |  |  | 42 |
| 361 |  |  |  | 41 |
| 372 |  |  |  | 40 |
| 398 |  |  | 4 |  |
| 405 |  |  |  | 38 |
| 406 |  |  |  | 37 |
| 424 |  |  |  | 35 |
| 427 |  |  |  | 34 |
| 433 |  |  |  | 33 |
| 436 |  |  |  | 31 |
| 449 |  |  |  | 30 |
| 450 |  |  |  | 29 |
| 456 |  |  |  | 28 |
| 462 |  |  |  | 27 |
| 465 |  |  |  | 26 |
| 471 |  |  |  | 24 |
| 475 |  |  |  | 22 |
| 485 |  |  |  | 21 |
| 488 |  |  |  | 20 |
| 496 |  |  |  | 19 |
| 514 |  |  |  | 18 |
| 522 |  |  |  | 16 |
| 528 |  |  |  | 14 |
| 539 |  |  |  | 13 |
| 560 |  |  |  | 12 |
| 606 |  |  |  | 10 |
| 634 |  |  |  | 9 |
| 667 |  |  |  | 7 |
| 700 | 3 | 3 | 3 | 5 |

^1^ Day after birth

**Supplementary Table 3.** Raw data for Figures 3 and 4

| **Patient ID** | **Polio adm.** | **Hib  adm.** | **Diphtheria  adm.** | **Tetanus  adm.** | **Polio  2 mo** | **Hib  2 mo** | **Diphtheria  2 mo** | **Tetanus  2 mo** | **Polio  12 mo** | **Hib  12 mo** | **Diphtheria  12 mo** | **Tetanus  12 mo** | **No of vaccinations at 12 mo** | **Probiotics^1^ in stool day 28-31** |
| --- | --- | --- | --- | --- | --- | --- | --- | --- | --- | --- | --- | --- | --- | --- |
| 12.1.8.011 | 15.9261 | 0.7168 | 0.0509 | 1.0394 |  |  |  |  | 11.3653 | 0.0200 | 0.0008 | 0.0214 | 0 | n.d. |
| 12.1.8.018 | 34.1096 | 0.0200 | 0.0236 | 0.6418 |  |  |  |  | 5.1310 | 0.0200 | 0.0009 | 0.0439 | 0 | n.d. |
| 12.2.8.013 | 13.1055 | 0.6314 | 0.0412 | 0.8208 |  |  |  |  | 6.7538 | 0.0910 | 0.0007 | 0.0193 | 0 | n.d. |
| 12.1.7.001 | 16.3443 | 1.8999 | 0.0873 | 0.9527 |  |  |  |  | 3.2583 | 0.0200 | 0.0316 | 0.1172 | 3 | yes |
| 12.1.7.002 | 22.5988 | 0.4287 | 0.0430 | 0.3313 |  |  |  |  | 5.7752 | 0.0874 | 0.0413 | 0.0897 | 3 | n.d. |
| 12.1.7.005 | 19.8436 | 0.4405 | 0.0418 | 0.1520 |  |  |  |  | 23.9505 | 3.4860 | 0.2368 | 2.6722 | 3^2^ | no |
| 12.1.7.012 | 10.0900 | 0.0200 | 0.0105 | 0.2042 | 3.9279 | 0.0200 | 0.0017 | 0.0314 | 8.9028 | 1.2815 | 0.5238 | 1.8425 | 3 | yes |
| 12.1.7.019 | 3.7962 | 0.7531 | 0.0034 | 0.1489 | 1.5983 | 0.2145 | 0.0011 | 0.0510 | 9.3429 | 0.4578 | 0.0533 | 1.0753 | 3 | yes |
| 12.1.7.024 | 8.4420 | 0.5361 | 0.0057 | 0.4800 | 2.7203 | 0.0697 | 0.0012 | 0.0660 | 1.7821 | 0.0200 | 0.0008 | 0.0310 | 3 | n.d. |
| 12.1.7.026 | 8.8098 | 0.1321 | 1.3723 | 1.0192 | 2.0089 | 0.0200 | 0.3263 | 0.1842 | 109.6235 | 0.4107 | 0.0692 | 1.6147 | 3^2^ | n.d. |
| 12.1.7.027 |  |  |  | 0.4767 |  |  |  |  |  |  |  | 0.0391 | 3 | yes |
| 12.1.8.001 | 22.2244 | 1.9580 | 0.6148 | 0.2567 |  |  |  |  | 71.6043 | 1.1277 | 0.1805 | 7.5953 | 3 | n.d. |
| 12.1.8.008 | 27.9503 | 0.0200 | 0.1614 | 0.3931 |  |  |  |  | 14.9338 | 1.1598 | 0.0343 | 0.0314 | 3 | yes |
| 12.1.8.009 | 21.1089 | 0.0200 | 0.1081 | 0.2338 |  |  |  |  | 11.7945 | 5.2877 | 0.0618 | 0.0364 | 3 | n.d. |
| 12.1.8.016 | 16.4433 | 1.0111 | 0.0469 | 0.4958 |  |  |  |  | 4.9246 | 0.4206 | 1.4814 | 0.9357 | 3 | yes |
| 12.2.7.001 | 18.6616 | 0.7855 | 0.4925 | 2.9411 | 6.3035 | 0.2671 | 0.1316 | 1.2006 | 30.7766 | 7.0528 | 0.0988 | 1.0886 | 3 | no |
| 12.2.7.003 | 41.1983 | 0.7121 | 0.0038 | 0.1211 | 7.4468 | 0.0667 | 0.0007 | 0.0162 | 210.8296 | 0.4138 | 0.1490 | 1.8025 | 3 | n.d. |
| 12.2.7.004 | 34.7308 | 0.5982 | 0.0063 | 0.1211 | 10.0251 | 0.1659 | 0.0013 | 0.0281 | 7.4495 | 0.5088 | 0.1729 | 4.3297 | 3 | n.d. |
| 12.2.7.009 | 11.9536 | 0.0286 | 0.0111 | 0.1911 | 2.5401 | 0.0200 | 0.0006 | 0.0060 | 3.7186 | 0.5633 | 0.5749 | 1.2694 | 3 | yes |
| 12.2.7.012 | 5.1458 | 1.0290 | 0.0049 | 0.2183 | 1.8604 | 0.3603 | 0.0041 | 0.0679 | 5.6887 | 0.1702 | 0.0312 | 0.1050 | 3 | yes |
| 12.2.7.015 | 2.9488 | 0.0200 | 0.0130 | 0.1702 |  |  |  |  | 10.6111 | 0.1061 | 0.0320 | 0.3370 | 3 | n.d. |
| 12.2.7.016 | 27.7314 | 0.6601 | 0.0440 | 0.1531 |  |  |  |  | 9.8089 | 0.4724 | 0.2380 | 0.6540 | 3 | yes |
| 12.2.7.017 | 8.8683 | 0.5785 | 0.0084 | 0.6268 | 3.9782 | 0.1901 | 0.0022 | 0.1550 | 319.3930 | 0.0200 | 0.0099 | 0.2960 | 3 | n.d. |
| 12.2.8.003 | 15.2704 | 0.6316 | 0.0108 | 0.2712 |  |  |  |  | 1.7594 | 0.3095 | 0.0660 | 0.1479 | 3 | n.d. |
| 12.2.8.005 | 5.4598 | 0.0893 | 0.1114 | 0.2436 |  |  |  |  | 11.6530 | 0.3879 | 0.3350 | 0.2244 | 3 | n.d. |
| 12.2.8.006 | 29.5941 | 0.0200 | 0.1444 | 0.3296 |  |  |  |  | 7.9360 | 5.6453 | 0.0408 | 0.0705 | 3 | yes |
| 12.2.8.007 | 22.7573 | 0.0200 | 0.1324 | 0.2870 |  |  |  |  | 13.3752 | 4.2837 | 0.0883 | 0.0481 | 3 | yes |
| 12.2.8.014 | 6.6764 | 0.1388 | 0.0101 | 0.0483 |  |  |  |  | 171.2285 | 5.7977 | 1.0337 | 7.8162 | 3 | yes |
| 12.2.8.015 | 11.2542 | 0.2371 | 0.0163 | 0.0439 |  |  |  |  | 283.0622 | 1.5531 | 0.6174 | 5.0086 | 3 | yes |
| 12.2.8.017 | 11.2111 | 0.4610 | 0.1881 | 1.4389 |  |  |  |  | 12.2345 | 1.1815 | 0.9516 | 0.1188 | 3 | n.d. |
| 12.2.8.021 |  |  |  | 3.2989 |  |  |  |  |  |  |  | 1.0438 | 3 | n.d. |
| 12.1.7.015 | 7.6230 | 0.6760 | 0.0426 | 0.2483 | 40.9033 | 0.0614 | 0.2339 | 0.0374 | 60.8250 | 15.00 | 2.7964 | 6.4442 | 4 | n.d. |
| 12.1.7.016 | 36.9875 | 0.5569 | 0.2186 | 0.2111 | 4.2584 | 0.0200 | 0.0218 | 0.0214 | 54.0632 | 15.00 | 2.8168 | 3.5550 | 4 | no |
| 12.1.7.017 | 4.9261 | 0.2491 | 2.9803 | 8.5976 | 0.9456 | 0.0200 | 0.4488 | 3.2126 | 68.4888 | 14.9062 | 1.1501 | 8.4464 | 4 | yes |
| 12.1.7.018 | 4.7392 | 0.0426 | 2.3953 | 7.7198 | 1.2264 | 0.0200 | 0.5760 | 2.4657 | 53.6009 | 15.00 | 1.5492 | 5.7237 | 4 | n.d. |
| 12.1.7.025 | 104.8959 | 0.2190 | 0.1820 | 0.8845 | 27.5270 | 0.0200 | 0.0455 | 0.2366 | 50.2761 | 1.6094 | 1.3879 | 1.1182 | 4 | n.d. |
| 12.1.8.010 | 6.8875 | 1.3185 | 0.1072 | 0.7546 |  |  |  |  | 95.7917 | 4.2943 | 4.2000 | 7.4841 | 4 | yes |
| 12.1.8.019 | 5.8788 | 0.3269 | 0.0695 | 0.2733 |  |  |  |  | 9.0570 | 11.1569 | 1.0186 | 1.5471 | 4 | n.d. |
| 12.2.7.011 | 33.6745 | 1.4768 | 0.0960 | 0.4276 | 9.0116 | 0.5992 | 0.0247 | 0.1310 | 40.5507 | 11.6898 | 3.3385 | 3.8782 | 4 | no |
| 12.2.7.013 | 2.8567 | 0.0200 | 0.1643 | 0.2162 | 0.8840 | 0.0200 | 0.0444 | 0.1119 | 60.3756 | 6.7512 | 1.3873 | 7.1594 | 4 | n.d. |
| 12.2.8.002 | 2.2325 | 0.1366 | 0.0149 | 0.0775 |  |  |  |  | 38.8246 | 0.4377 | 4.1939 | 11.3976 | 4 | yes |
| 12.2.8.016 | 11.6806 | 0.7608 | 0.0040 | 0.1827 |  |  |  |  | 14.5809 | 4.3963 | 3.1956 | 2.0888 | 4 | n.d. |
| 12.2.8.020 | 1.3121 | 0.4045 | 0.7526 | 0.3349 |  |  |  |  | 24.5383 | 8.5514 | 1.5343 | 2.9595 |  | yes |

# ^1^ Bifidobacterium animalis subsp. lactis, B. infantis and/or Lactobacillus acidophilus

# ^2^ fourth vaccination less than five days before blood sampling

## Supplementary Figure

**
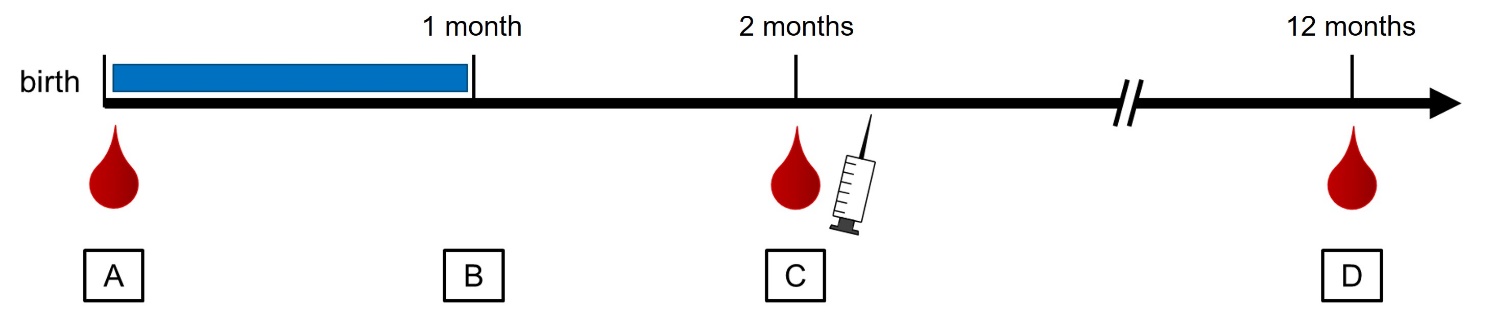
**

**Supplementary Figure 1** Sampling schedule for the present trial as part of the PRIMAL study [Marißen et al. 2019, Van Rossum et al. 2024]. Blood samples were collected [A] after admission (median 3 days of life, IQR 1 day), [C] two months after birth prior to the first hexavalent vaccination (median 56 days of life, IQR 10 days) and [D] at one year follow up = corrected age of approximately 12 months (median 437 days of life, IQR 24 days). Stool samples for microbiome analysis were collected [B] at one month (median 30 days of life, IQR 2 days). The PRIMAL trial intervention (blue bar) started as soon as possible after randomization with daily enteral administration of the probiotic mix (Bifidobacterium longum, B. infantis and Lactobacillus acidophilus) or placebo over 28 days.

References to Supplementary Figure 1:

• Marissen J, Haiss A, Meyer C, et al. Efficacy of Bifidobacterium longum, B. infantis and Lactobacillus acidophilus probiotics to prevent gut dysbiosis in preterm infants of 28+0-32+6 weeks of gestation: a randomised, placebo-controlled, double-blind, multicentre trial: the PRIMAL Clinical Study protocol. BMJ Open. 2019;9:e032617 doi: 10.1136/bmjopen-2019-032617

• Van Rossum T, Haiss A, Knoll RL, et al. Bifidobacterium and Lactobacillus Probiotics and Gut Dysbiosis in Preterm Infants: The PRIMAL Randomized Clinical Trial. JAMA Pediatr. 2024;178:985-95 doi: 10.1001/jamapediatrics.2024.2626
